# Susceptibility to Nisin, Bactofencin, Pediocin and Reuterin of Multidrug Resistant *Staphylococcus aureus*, *Streptococcus dysgalactiae* and *Streptococcus uberis* Causing Bovine Mastitis

**DOI:** 10.3390/antibiotics10111418

**Published:** 2021-11-19

**Authors:** Samantha Bennett, Laila Ben Said, Pierre Lacasse, François Malouin, Ismail Fliss

**Affiliations:** 1 Sherbrooke Research and Development Centre, Agriculture and Agri-Food Canada, Sherbrooke, QC J1M 0C8, Canada; samantha.bennett@usherbrooke.ca (S.B.); pierre.lacasse@canada.ca (P.L.); 2 Département de Biologie, Faculté des Sciences, Université de Sherbrooke, Sherbrooke, QC J1K 2R1, Canada; francois.malouin@usherbrooke.ca; 3 Food Science Department, Food and Agriculture Faculty, Institute of Nutrition and Functional Foods, Laval University, 2425 Agriculture Street, Quebec City, QC G1V 0A6, Canada; ismail.fliss@fsaa.ulaval.ca

**Keywords:** antibiotic resistance, bacteriocin, dairy cows, intramammary infection, alternative to antibiotics, reuterin

## Abstract

Antibiotics are the most effective strategy to prevent and treat intramammary infections. However, their misuse has led to the dissemination of multidrug resistant bacteria (MDR) for both animals and humans. Efforts to develop new alternative strategies to control bacterial infections related to MDR are continuously on the rise. The objective of this study was to evaluate the antimicrobial activity of different bacteriocins and reuterin against MDR *Staphylococcus* and *Streptococcus* clinical isolates involved in bovine mastitis. A bacterial collection including *S. aureus* (*n* = 19), *S. dysgalactiae* (*n* = 17) and *S. uberis* (*n* = 19) was assembled for this study. Antibiotic resistance profiles were determined by the disk diffusion method. In addition, sensitivity to bacteriocins and reuterin was evaluated by determining minimum inhibitory concentrations (MIC). A total of 21 strains (37.5%) were MDR. MICs ranged from ≤1.0 μg/mL to ≥100 μg/mL for nisin and 2.0 to ≥250 μg/mL for bactofencin. Reuterin was active against all tested bacteria, and MICs vary between 70 and 560 μg/mL. Interestingly, 20 MDR strains were inhibited by bactofencin at a concentration of ≤250 μg/mL, while 14 were inhibited by nisin at an MIC of ≤100 μg/mL. Pediocin did not show an inhibitory effect.

## 1. Introduction

The discovery of antibiotics in the 20th century is attributed to the evolution of modern medicine. Over the years, this scientific advancement contributed to saving millions of lives as well as controlling infectious diseases [1]. However, the misuse and overuse of antibiotics has led to the rapid emergence of antibiotic-resistant bacteria, which have become an alarming and growing public health concern worldwide. Conventional antibiotics are becoming less effective, and few new antibiotic classes are being discovered. Consequently, numerous infectious diseases have become harder and sometimes impossible to treat [2,3,4].

According to the Centers for Disease Control and Prevention (CDC), in the United-States, there are over 2.8 million antibiotic-resistant infections causing over 35,000 deaths every year [1]. The extensive use of antibiotics in both human medicine and agriculture is known to have contributed to the crisis [5,6]. Although decades of misuse of antibiotics in human medicine has had a major impact, reducing antimicrobials in agriculture has been the main strategy in reducing the spread of resistance, partially due to the use of similar drugs in both human and animal infections [7]. For these reasons, in the last decade, many countries have implemented strict regulations to reduce and control antibiotic utilization in animal production.

Bovine mastitis is one of the most persistent and costly diseases affecting dairy cattle worldwide [8]. This disease leads to significant economic consequences caused by milk production loss, cost of treatment, discarded milk, and veterinary expenses, among other factors [9,10,11]. Bovine mastitis can be caused by many microorganisms, of which *Staphylococcus aureus*, *Streptococcus dysgalactiae*, and *Streptococcus uberis* are among the most common. This infection is most often treated with antibiotics, and is the leading cause of antimicrobial usage in the dairy industry. Although effective, the use of antibiotics in the dairy industry presents many disadvantages, such as leaving residues in milk. Therefore, it has become important to develop novel alternatives in order to reduce the spread of resistance while controlling animal infections.

Among currently studied therapeutic alternatives, bacteriocins have shown promising potential. Bacteriocins are antimicrobial substances of a proteinaceous nature which are ribosomally synthesized by a wide variety of bacteria. They act as a defense line for producing strains by inhibiting growth or killing other microorganisms in their competitive environments [12]. As opposed to antibiotics, most bacteriocins have a narrow spectrum of antimicrobial activity [13], which give the advantage of being able to target specific pathogenic organisms. Various bacteriocins have been identified, extensively characterized and described in the open-access database BACTIBASE [14], available at http://bactibase.hammamilab.org (accessed on 3 November 2021). While their main application is the control of foodborne pathogens for food preservation, their potential in treating human and animal infections has also been shown [15,16,17].

Despite these few conclusive data on the potential of bacteriocins in the treatment and prevention of bovine mastitis, no systematic study has been conducted to assess the inhibitory activity of different Gram-positive bacteriocins against multidrug resistant (MDR) microorganisms responsible for bovine mastitis. Moreover, determining the extent of the spectrum of inhibition of each bacteriocin as well as their mechanism of action (bactericidal or bacteriostatic) will allow more effective and better targeted treatments for bovine mastitis to be developed. This information will also lead to the development of original strategies based on the use of several bacteriocins in rotation or in synergistic consortia to broaden the spectrum of action and limit the development of resistance to these bacteriocins [18,19,20].

Hundreds of bacteriocins produced by Gram-positive bacteria have been described in the literature. Some are well characterized, while others remain very little studied. One of the least studied aspects is the spectrum of inhibition of these bacteriocins. Nisin A, a lantibiotic produced by *Lactococcus lactis*, shows antimicrobial activity against a wide range of Gram-positive bacteria [21,22]. Its mechanism of action is based on the disruption of the bacterial cell wall by the formation of pores as well as the inhibition of peptidoglycan precursors. Pediocin PA-1 is produced by Gram-positive *Pediococcus acidilactici* and exhibits inhibitory activity against *Listeria monocytogenes* and *L. ivanovii* by forming pores in the cytoplasmic membrane of target cells [23]. Bactofencin A is isolated from Gram-positive *Lactobacillus salivarius* [24] and has shown inhibitory activity against both *S. aureus* and *L. monocytogenes* by targeting bacterial cell wall components [25]. Bactofencin A is a novel cationic peptide, the mechanism of action of which seems relatively unique [26]. Its antimicrobial activity is based on the modification of teichoic acids, a component of the cell wall, causing its disruption [27].

Reuterin is an antimicrobial aldehyde produced by *Lactobacillus reuteri* and is known to induce oxidative stress in cells by modifying thiol groups in proteins [28,29,30]. Its mechanism of action is not specific to a cell type; therefore, reuterin shows antimicrobial activity against a broad range of Gram-positive and Gram-negative bacteria, as well as fungi, yeast and certain viruses [31,32].

Thus, the present study aimed to carry out a systematic study to qualitatively and quantitatively evaluate and characterize the antimicrobial activity of different Gram-positive bacteriocins against a large panel of MDR clinical staphylococci and streptococci isolates.

## 2. Results and Discussions

### 2.1. Antimicrobial Compound Production and Purification

**Figure 1:**
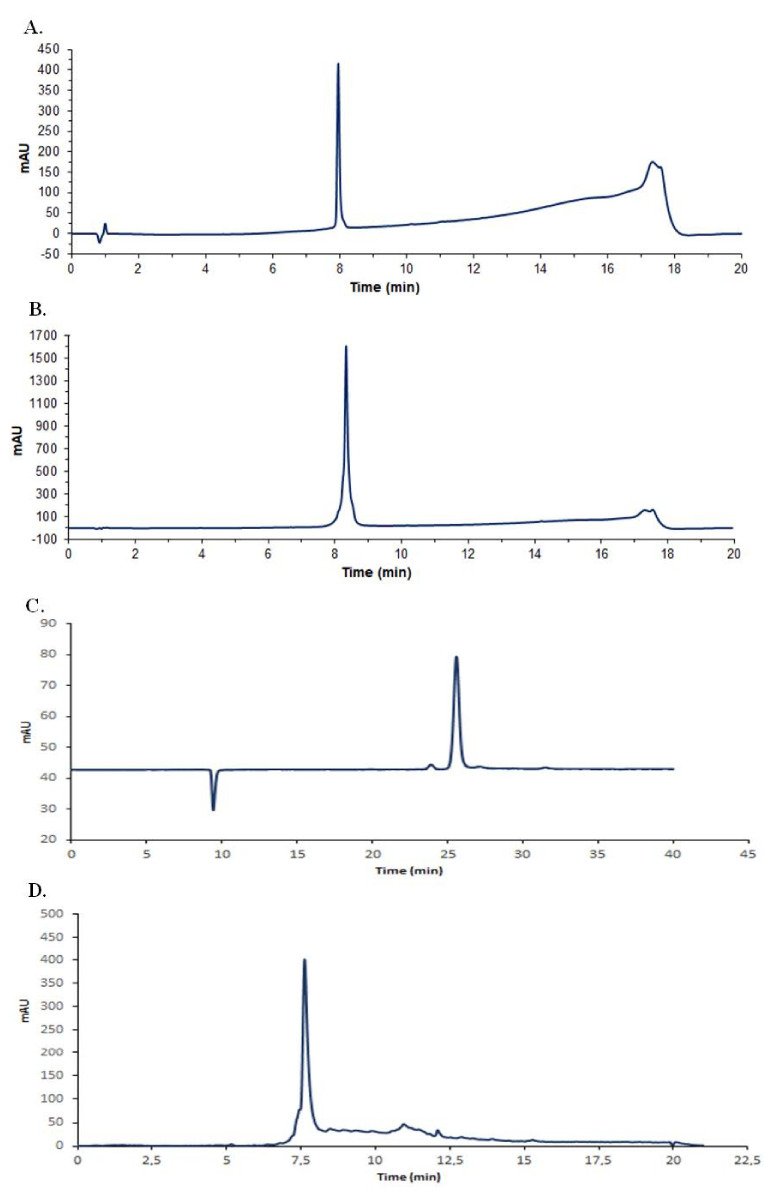
HPLC chromatogram profiles of purified antimicrobials. (**A**), bactofencin; (**B**), pediocin PA-1; (**C**), reuterin and (**D**), nisin, where mAU is the intensity of absorbance.

Bactofencin A and pediocin PA-1 were successfully synthesized with high purity (Figure 1A,B). The concentration of reuterin produced from the bioconversion of glycerol reached 200 mmol/L, which corresponded to a yield of 92% (Figure 1C). Nisin was purified and reached a purity higher than 90% (Figure 1D).

### 2.2. Antibiotic Susceptibility Profiles

The agar disk diffusion assay revealed several antibiotic susceptibility profiles. Overall, among the 55 isolates, 34 (62%) were resistant to at least one antibiotic and 21 (38%) were MDR (resistant to 3 or more antibiotic classes). More precisely, among the *S. aureus* (*n* = 19) isolates, 10 were resistant to at least one antibiotic and 6 were MDR. Similarly, among the *S. dysgalactiae* (*n* = 17) and *S. uberis* (*n* = 19) isolates, 13 and 11 were resistant to at least one antibiotic, and 8 and 7 were MDR, respectively (Table 1).

For all bacterial groups, the highest rate of resistance was observed with penicillin and amoxicillin-clavulanic acid, as reported in other studies [33,34,35], while resistance rates to ciprofloxacin and vancomycin were low [36,37]. Moreover, all strains were sensitive to clindamycin, unlike previous studies, where greater resistance rates have been observed [37,38]. In the present study, the isolates showed higher resistant rates to cephalothin, cefotaxime and cefoxitin than those reported by others [33,39]. Low resistance rates to the penicillin-novobiocin combination were observed as reported in accordance with previous studies [39,40]. Indeed, combination therapies including penicillin-novobiocin are commonly used to treat and prevent intramammary infections. It is well known that combination therapies reduce the risk of resistance, broaden the spectrum of activity and potentially enhance antimicrobial activity with an additive of synergistic activity [41]. As expected, higher resistance rates to penicillin were observed in comparison to the penicillin-novobiocin combination. Here, by determining the antibiotic susceptibility profiles of various clinical strains, it was possible to demonstrate the potential of bacteriocins against strains inclined to be encountered in herds, which include MDR strains.

For decades, antibiotics have been used to treat microbial infections in dairy cattle, and the widespread use of penicillins as well as cephalosporines is still common [42]. The use of sub-lethal concentrations has been thought to have gradually induced antibiotic resistance by pressure selection [43,44]. Despite the best choice of treatment, antimicrobial resistance is implicated in failure of treatment, notably for *S. aureus* [45]. Worldwide, resistance to ß-lactams is prevalent in clinical isolates, both human and animal, and there is a correlation between antimicrobial usage and antimicrobial resistant bacteria in agriculture [46,47]. Despite the emergence of antibiotic-resistant pathogens, there has been a lack of development of new antibiotics. It has become urgent to develop alternatives to antibiotics with different mechanisms of action in order to control infectious diseases in both humans and animals.

### 2.3. Antimicrobial Activity

**Figure 2:**
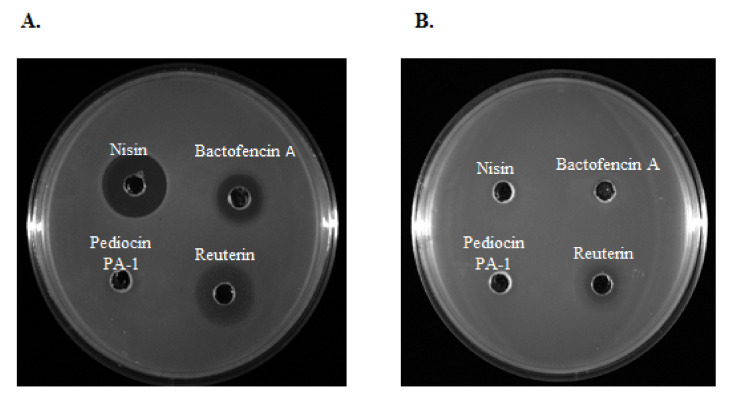
The antimicrobial activity of nisin (250 μg/mL), bactofencin A (250 μg/mL), pediocin PA-1 (250 μg/mL) and reuterin (3.7 mg/mL) against MDR (**A**) *Staphylococcus aureus* 40709611 [PEN-CEF-FOX-CTX-ERY-CHL-KAN-GEN-PEN/NOV] and (**B**) *Staphylococcus aureus* 40410425 [VAN-FOX-CTX-ERY-CHL-TET-KAN-GEN].

The antimicrobial activity of bacteriocins and reuterin was first assessed by radial diffusion assays with *Staphylococcus* and *Streptococcus* isolates from clinical bovine mastitis. Results show that bactofencin, nisin and reuterin were active against all isolates sensitive to antibiotics. Interestingly, bactofencin (*n* = 20; 95%), nisin (*n* = 14; 67%) and reuterin (*n* = 21, 100%) displayed high antimicrobial activity against certain MDR isolates (Figure 2A). However, a few MDR strains were co-resistant to nisin and bactofencin and less sensitive to reuterin (Figure 2B). These results demonstrate the possibility of cross-resistance between conventional antibiotics and bacteriocins.

#### 2.3.1. Antimicrobial Activity of Nisin

In the present study, nisin showed inhibitory activity against 48 strains (87.2%), including *S. aureus*, *S. dysgalactiae* and *S. uberis*. MIC values ranged between ≤1.0 and ≥100 μg/mL (Table 2). For each bacterial group, MBC values were one, two or four folds above the MIC concentrations; therefore, nisin presents a bactericidal activity.

Seven strains, including one *S. aureus*, two *S. dysgalactiae* and four *S. uberis*, were not inhibited at a concentration of 100 μg/mL. Interestingly, all seven strains were multi-resistant to antibiotics. The phenomena of cross-resistance between nisin and antibiotics is possible and requires further investigation. Antimicrobial activity of nisin against a broad-range of Gram-positive bacteria has been previously reported [48,49,50]. In agreement with previous studies, nisin showed antimicrobial activity against MDR pathogens [48,51,52].

#### 2.3.2. Antimicrobial Activity of Reuterin

Reuterin was active against all isolates, including both those which were susceptible and those which were resistant to classical antibiotics. The MICs of reuterin against all tested strains varied between 0.07 mg/mL and 0.56 mg/mL regardless of the species (Table 3). Values of MBC for *S. dysgalactiae* and *S. uberis* were one, two or four times the MIC, indicating that reuterin exhibited a bactericidal effect against streptococci species. However, higher concentrations of reuterin were necessary to provide bactericidal activity against *S. aureus*, with an MBC value that varied between 8 and 32 times the MIC. Hence, reuterin seems to be bacteriostatic against *S. aureus.* Overall, antibiotic resistance did not affect MIC and MBC values.

To our knowledge, this is the first report of purified reuterin’s antimicrobial activity against a collection of mastitis-causing pathogens with the final goal of preventing or treating intramammary infections in dairy cows. In accordance with our results, Chen et al. [53] previously reported the antimicrobial activity of reuterin against *S. aureus*. Furthermore, studies have demonstrated that reuterin or *L. reuteri* was active against certain *Streptococcus* species, such as *Streptococcus salivarius* [32] and *Streptococcus lactis* [31]. Unfortunately, it is difficult to compare results between different studies, as the antimicrobial activity of reuterin is often presented as arbitrary units. Interestingly, Arqués et al. [54] revealed that reuterin is capable of causing growth inhibition of *S. aureus* in milk for 24 h at 37 °C. Reuterin is a natural compound that shows promise in treating bovine mastitis. In addition to its antimicrobial activity, reuterin is known for its decontamination properties in the food industry to control foodborne pathogens [55]. Unfortunately, most studies implicating reuterin focus on the probiotic properties of *L. reuteri*. Nevertheless, our results indicate that reuterin is active against *Staphylococcus aureus*, *Streptococcus dysgalactiae* and *Streptococcus uberis* causing mastitis. Further studies should investigate the efficacy of purified reuterin in treating bovine mastitis, as well as its safety on the mammary gland and other tissues.

#### 2.3.3. Antimicrobial Activity of Bactofencin A

Bactofencin A showed antimicrobial activity against antibiotic-susceptible and MDR isolates. Lower concentrations of the peptide were needed to inhibit the growth of *S. aureus* isolates in comparison to streptococci species. Indeed, MIC50 values for *S. aureus*, *S. dysgalactiae* and *S. uberis* were 3.9 μg/mL, 62.5 μg/mL and 15.6 μg/mL, respectively. Higher concentrations of bactofencin A were needed to exhibit a bactericidal effect, and MBC50 values for *S. aureus*, *S. dysgalactiae* and *S. uberis* were 31.2 μg/mL, 125 μg/mL and 31.2 μg/mL, respectively (Table 4). Thus, this peptide exhibited a bacteriostatic effect against *S. aureus* and a bactericidal effect against streptococci species. Only one MDR *S. aureus* strain was not inhibited with concentrations reaching 250 μg/mL; otherwise, the MIC values required to inhibit antibiotic-susceptible strains were comparable to those inhibiting strains resistant to classical antibiotics.

Bactofencin A was expected to be highly active against *S. aureus*, as other studies have reported anti-Listeria and anti-*S. aureus* activity [25,27]. To our knowledge, this is the first evidence that bactofencin A is also active against *S. dysgalactiae* and *S. uberis*. In accordance with other studies, this peptide was active against MDR isolates [25]. These authors recently investigated the presence of cross-resistance between bactofencin and antibiotics by comparing MIC values of bactofencin against methicillin-susceptible and methicillin-resistant *S. aureus* isolates. In accordance with our study, their results showed a lack of cross-resistance between both groups.

#### 2.3.4. Antimicrobial Activity of Pediocin

Our results demonstrated that pediocin was not active against the tested isolates even at the highest concentration tested (500 μg/mL). Pediocin is known for its antimicrobial activity against the Gram-positive foodborne pathogen *Listeria monocytogenes* [24,56]. In accordance with the results obtained in the present study, a previous work has shown that purified pediocin PA-1 did not inhibit growth of *S. aureus* [57]. The mechanism of action of pediocin PA-1 is similar to that of nisin. Indeed, pediocin’s antimicrobial activity is based on its nonspecific adhesion to the cytoplasmic membrane, followed by binding specifically to a receptor-like molecule present on the surface. The peptide then inserts into the host cell, forming pores in the membrane which cause a release of ions and molecules leading to cell death [23].

Moreover, it has been demonstrated that class IIa bacteriocins, including pediocin PA-1, interact with the mannose phosphotransferase system (Man-PTS) [58,59]. More recently, a sugar transporting system was shown to be involved in several intracellular processes [60]. Indeed, various studies revealed that resistant strains to class IIa bacteriocins present lower Man-PTS gene expression [59,61]. This could explain its narrow spectrum of action compared to nisin. Hence, the potential of this peptide in treating bovine mastitis appears low.

## 3. Materials and Methods

### 3.1. Bacterial Strains

A collection of *Staphylococcus aureus* (*n* = 19), *Streptococcus dysgalactiae* (*n* = 17) and *Streptococcus uberis* (*n* = 19) isolated from clinical intramammary infections (IMI) were selected from the Mastitis Pathogen Culture Collection of the Canadian Bovine Mastitis Network (Université de Montréal, St-Hyacinthe, QC, Canada). The pathogens were isolated from milk samples in dairy cows with clinical mastitis as described by Reyher et al. [62], where milk samples of infected quarters were collected on dairy cows showing clinical signs or abnormal milk. Identification of the isolates was confirmed by MALDI-TOF mass spectrometry. The isolates were received in lyophilized form, washed in 0.9% NaCl aqueous solution and conserved in brain heart infusion broth (BHIB, Becton, Dickinson-Difco, Sparks, MD, USA) containing 20% glycerol at −80 °C until further use.

### 3.2. Antimicrobial Compound Production and Purification

Pediocin PA-1 and bactofencin A were chemically synthesized and purified as described by Bédard, Hammami, Zirah, Rebuffat, Fliss and Biron [24] and Bédard, Fliss and Biron [25]. HPLC-MS was performed on a Shimadzu Prominence LC/MS-2020 system prepped with an electrospray ionization probe using a Kinetex column (4.6 mm × 100 mm, 2.6 μm XB-C18, 100 Å, 1.4 mL/min). Elution was performed with a gradient from water (0.1% HCOOH) and CH_3_CN (0.1% HCOOH, 10 to 100% ${\mathrm{CH}}_{3}\mathrm{CN}$).

Purification of nisin was based on a method described by Gough et al. [63] with some modifications. In essence, 25 g of commercial nisin powder (Siveele, Breda, the Netherlands) was dissolved in 500 mL of milliQ water. The solution was centrifuged three times at 14,000× *g* at 18 ${}^{\circ }$C for 15 min. After each centrifugation, the supernatant was discarded, and the pellet was dissolved in a smaller volume of milliQ water (500, 250, 125 mL). The final pellet was dissolved in 60 mL of milliQ water, and the solution was filtered through a 0.22 μm filter (Sartedt Inc., Nümbrecht, Germany). To assure the obtained fraction contained purified nisin, the fraction was analyzed by reverse-phase HPLC on a C18 column (AerisTM 3.6 μm, PEPTIDE XB-C18 250 × 4.6 mm). Production and purification of reuterin was performed as described by Vimont et al. [64]. HPLC chromatogram of purified reuterin was obtained using a Coregel ION-300 column (7.8 × 300 mm, sulfonated polystyrene/divinylbenzene copolymers). Elution was performed with 10 mM H${}_{2}$SO${}_{4}$ with a flow rate of 0.4 mL/min. All bacteriocins and reuterin preparations were maintained at high concentrations as stock solutions and stored at −20 ${}^{\circ }$C until use.

Antimicrobial activity of nisin, bactofencin A, pediocin PA-1 and reuterin was demonstrated using an agar diffusion assay on *Staphylococcus aureus* 32013313 and *Staphylococcus aureus* 40410425, as previously described [65]. Briefly, 75 μL of the purified antimicrobial peptides were added in wells on TSA soft agar (0.75% *w*/*v*) seeded with the *S. aureus* strains. Plates were incubated at 37 ${}^{\circ }$C for 18 h and antimicrobial activity. The inhibition zones revealed the antimicrobial activity of the peptides against the given strain.

### 3.3. Antibiotic Susceptibility Testing

Susceptibility of the different isolates to several antibiotics was determined using the disc diffusion method according to the Clinical and Laboratory Standards Institute’s guidelines [66]. Bacteria were grown in BHIB for 18 h at 37 ${}^{\circ }$C and then diluted in BHIB to obtain a suspension of approximately $10^{6}$ cfu/mL. Then, 1 mL of the bacterial suspension was spread on Mueller–Hinton agar (MHA, BD-Difco, Alberta, AB, Canada) supplemented with 5% (*v*/*v*) sheep blood for *Streptococcus*. Plates were incubated at 37 ${}^{\circ }$C in aerobic conditions. For quality control, *Staphylococcus aureus* ATCC 25925 with a known antibiotic resistance profile was used as a reference strain. The following antibiotics were tested: penicillin (10 U), amoxicillin/clavulanic acid (30 μg), vancomycin (30 μg), cephalothin (30 μg), cefoxitin (30 μg), cefotaxime (30 μg), erythromycin (15 μg), chloramphenicol (2 μg), clindamycin (30 μg), ciprofloxacin (5 μg), novobiocin (30 μg), penicillin-novobiocin (30 μg) and tetracycline (30 μg) (ThermoFisher, Waltham, MA, USA). For *S. aureus* strains only, kanamycin (30 μg) and gentamicin (10 μg) were also tested. Penicillin-novobiocin combination disks were prepared as described by Thornsberry, Burton, Yee, Watts and Yancey [40] the day of utilization. Interpretation of susceptibility patterns were performed according to the Clinical and Laboratory Standard Institute [66].

### 3.4. Minimum Inhibitory and Bactericidal Concentrations

Antimicrobial susceptibility of the isolates to bactofencin A, nisin, pediocin PA-1 and reuterin was evaluated by a microdilution method. Assays were performed in 96-well microtiter plates in accordance with the guidelines established by the Clinical and Laboratory Standard Institute [63]. Strains were grown in BHIB, and bacteria were diluted to obtain a suspension of 5 × 10${}^{5}$ cfu/mL. A volume of 50 μL of the suspension was added to each well, excluding the negative control. Antimicrobial compounds were added by serial two-fold dilutions, and the following concentrations of antimicrobial compounds were used: bactofencin A (0.5–250 μg/mL), nisin (0.1–100 μg/mL), pediocin PA-1 (0.4–500 μg/mL) and reuterin (4.4–2240 μg/mL). Plates were incubated aerobically at 37 ${}^{\circ }$C for 18 h. The minimal inhibitory concentration (MIC) was defined as the lowest concentration that inhibited visual growth. The minimal bactericidal concentration (MBC) was determined by inoculating an MH agar surface with 10 μL from wells showing complete inhibition and incubating for 24 h at 37 ${}^{\circ }$C. Antimicrobial activity can be classified into two groups: bacteriostatic (MBC/MIC > 4) and bactericidal (MBC/MIC ≤ 4). A bacteriostatic compound is capable of inhibiting bacterial growth, whereas a bactericidal effect is the capacity to kill bacteria [67]. The MBC was the concentration killing 99.9% of the initial inoculum. To assure quality control, *S. aureus* ATCC 25923 and *S. aureus* ATCC 6538 were used as reference strains. MIC determination was done in triplicate on independent plates.

## 4. Conclusions

The aim of this study was to evaluate the antimicrobial activity of several natural compounds against both susceptible and MDR mastitis-causing pathogens with the intent of using bacteriocins as an alternative to antibiotics for treating bovine mastitis. Our results demonstrated that bactofencin, nisin and reuterin are active against MDR clinical bovine mastitis isolates. Therfore, they show promise to be used as an alternative in reducing the use of antibiotics in animal production. Further studies on dairy cows should be performed in order to evaluate their safety as well as their efficacy in treating and preventing bovine mastitis. 

## Figures and Tables

**Table 1:** Antibiotic resistance profile of *Staphylococcus aureus*, *Streptococcus dysgalactiae* and *Streptococcus uberis* isolated from clinical bovine mastitis.

	*S. aureus* (*n* = 19)	*S. dysgalactiae* (*n* = 17)	*S. uberis* (*n* = 19)
	No. of Resistant Strains (%)		
PEN	9 (50)	10 (59)	9 (50)
P/N	1 (6)	3 (18)	5 (28)
AMC	5 (28)	8 (47)	5 (28)
VAN	2 (11)	2 (12)	4 (22)
CEF	7 (39)	9 (53)	7 (39)
CTX	7 (39)	9 (53)	6 (33)
FOX	7 (39)	10 (59)	6 (33)
ERY	3 (17)	4 (24)	5 (28)
CHL	5 (28)	6 (35)	5 (28)
CIP	2 (11)	0 (0)	0 (0)
CC	0 (0)	0 (0)	0 (0)
TET	3 (17)	5 (29)	2 (11)
KAN	5 (28)	-	-
GEN	4 (22)	-	-
MDR	6 (32)	8 (47)	7 (37)

No. (%), number and percentage of resistant isolates; PEN, penicillin; P/N, penicillin/novobiocin; AMC, amoxicillin/clavulanic acid; VAN, vancomycin; CEF, cephalothin; CTX, cefotaxime; FOX, cefoxitin; ERY, erythromycin; CHL, chloramphenicol; CIP, ciprofloxacin; CC, clindamycin; TET, tetracycline; KAN, kanamycin; GEN, gentamicin. “-”, not tested.

**Table 2:** MIC and MBC intervals, as well as MIC50 and MBC50 (μg/mL) values, of nisin against *Staphylococcus* and *Streptococcus* causing mastitis.

Species (*n*)	AMR ${}^{1}$	No. ${}^{2}$	MIC50 ${}^{3}$	MIC Interval	MBC50 ${}^{4}$	MBC Interval	Ratio ${}^{5}$	N ${}^{6}$
*S. aureus* (19)		19	7.8	2.0–≥100	15.6	3.9–≥100	2	1
	0	9	7.8	2.0–15.6	15.6	3.9–31.2	2	0
	1 or 2	4	7.8	3.9–7.8	15.6	7.8–31.2	2	0
	≥3	6	7.8	3.9–≥100	15.6	7.8–≥100	2	1
*S. dysgalactiae* (17)		17	7.8	1.0–≥100	15.6	1.0–≥100	2	2
	0	4	7.8	7.8–15.6	15.6	7.8–31.2	2	0
	1 or 2	5	7.8	7.8–15.6	7.8	7.8–15.6	1	0
	≥3	8	15.6	1.0–≥100	15.6	1.0–≥100	1	2
*S. uberis*(19)		19	3.9	≤1.0–≥100	7.8	≤1.0–≥100	2	4
	0	8	3.9	≤1.0–7.8	15.6	2.0–15.6	4	0
	1 or 2	4	1.0	≤1.0–3.9	2.0	≤1.0–3.9	2	0
	≥3	7	≥100	3.9–≥100	≥100	3.9–≥100	N/D ${}^{7}$	4

${}^{1}$ AMR, category of antimicrobial resistance: 0, 1–2 or 3 antibiotic classes for which bacterial strains are resistant. ${}^{2}$ No, number of bacterial strains in each category of AMR. ${}^{3}$ MIC50, minimal inhibitory concentration for 50% of the isolates. ${}^{4}$ MBC50, minimal bactericidal concentration for 50% of the isolates. ${}^{5}$ Ratio, MBC50/MIC50. ${}^{6}$ N, number of isolates where growth was not inhibited at the highest concentration tested (100 μg/mL). ${}^{7}$ N/D, not determined.

**Table 3:** MIC and MBC intervals, as well as MIC50 and MBC50 (μg/mL) values, of reuterin against *Staphylococcus* and *Streptococcus* causing mastitis.

Species (*n*)	AMR ${}^{1}$	No. ${}^{2}$	MIC50 ${}^{3}$	MIC Interval	MBC50 ${}^{4}$	MBC Interval	Ratio ${}^{5}$	N ${}^{6}$
*S. aureus* (19)		19	140	70–560	1120	560–2240	8	0
	0	9	70	70–560	1120	560–2240	16	0
	1 or 2	4	140	140	1120	560–1120	8	0
	≥3	6	280	70–560	1120	560–2240	4	0
*S. dysgalactiae* (17)		17	140	70–560	280	140–2240	2	0
	0	4	140	70–560	560	140–1120	4	0
	1 or 2	5	140	140	140	140–2240	1	0
	≥3	8	280	140–560	560	140–2240	2	0
*S. uberis* (19)		19	280	140–560	280	280–2240	1	0
	0	8	280	140–280	280	280–560	1	0
	1 or 2	4	280	140–280	280	280–560	1	0
	≥3	7	280	140–560	1120	280–2240	4	0

${}^{1}$ AMR, category of antimicrobial resistance: 0, 1–2 or 3 antibiotic classes for which bacterial strains are resistant. ${}^{2}$ No, number of bacterial strains in each category of AMR. ${}^{3}$ MIC50, minimal inhibitory concentration for 50% of the isolates. ${}^{4}$ MBC50, minimal bactericidal concentration for 50% of the isolates. ${}^{5}$ Ratio, MBC50/MIC50. ${}^{6}$ N, number of isolates where growth was not inhibited at the highest concentration tested (2240 μg/mL).

**Table 4:** MIC and MBC intervals, as well as MIC50 and MBC50 (μg/mL) values, of bactofencin against *Staphylococcus* and *Streptococcus* causing mastitis.

Species (n)	AMR ${}^{1}$	No. ${}^{2}$	MIC50 ${}^{3}$	MIC Interval	MBC50 ${}^{4}$	MBC Interval	Ratio ${}^{5}$	N ${}^{6}$
*S. aureus* (19)		19	3.9	2.0–≥250	31.2	15.6–≥250	8	1
	0	9	2.0	2.0–7.8	31.2	15.6–250	16	0
	1 or 2	4	3.9	3.9–15.6	62.5	31.2–125	16	0
	≥3	6	7.8	2.0–≥250	62.5	31.2–≥250	8	1
*S. dysgalactiae* (17)		17	62.5	7.8–62.5	125	31.2–125	2	0
	0	4	62.5	31.2–62.5	125	125	2	0
	1 or 2	5	62.5	62.5	125	125	2	0
	≥3	8	62.5	7.8–62.5	125	31.2–125	2	0
*S. uberis* (19)		19	15.6	3.9–62.5	31.2	15.6–62.5	2	0
	0	8	15.6	3.9–15.6	31.2	15.6–62.5	2	0
	1 or 2	4	15.6	15.6	31.2	31.2–62.5	2	0
	≥3	7	15.6	7.8–62.5	31.2	31.2–62.5	2	0

${}^{1}$ AMR, category of antimicrobial resistance: 0, 1–2 or 3 antibiotic classes for which bacterial strains are resistant. ${}^{2}$ No, number of bacterial strains in each category of AMR. ${}^{3}$ MIC50, minimal inhibitory concentration for 50% of the isolates. ${}^{4}$ MBC50, minimal bactericidal concentration for 50% of the isolates. ${}^{5}$ Ratio, MBC50/MIC50. ${}^{6}$ N, number of isolates where growth was not inhibited at the highest concentration tested (250 μg/mL).

## Data Availability

The data presented in this study are available on request from the corresponding author.
